# The New Zealand 1986 very low birth weight cohort as young adults: mapping the road ahead

**DOI:** 10.1186/s12887-015-0413-9

**Published:** 2015-08-05

**Authors:** Brian A. Darlow, L. John Horwood, Lianne J. Woodward, John M. Elliott, Richard W. Troughton, Mark J. Elder, Michael J. Epton, Josh D. Stanton, Maureen P. Swanney, Ross Keenan, Tracy R. Melzer, Victoria A. McKelvey, Karelia Levin, Margaret G. Meeks, Eric A. Espiner, Vicky A Cameron, Julia Martin

**Affiliations:** Cure Kids Professor of Paediatric Research, Department of Paediatrics, University of Otago at Christchurch, PO Box 4345, Christchurch, 8140 New Zealand; Christchurch Health and Development Study, Department of Psychological Medicine, University of Otago at Christchurch, PO Box 4345, Christchurch, 8140 New Zealand; Director of Infant and Child Development Research, Department of Pediatric Newborn Medicine. Brigham & Women’s Hospital, Harvard Medical School, Boston, MA 02115 USA; Christchurch Heart Institute, University of Otago at Christchurch, PO Box 4345, Christchurch, 8140 New Zealand; Department of Surgery, University of Otago at Christchurch, PO Box 4345, Christchurch, 8140 New Zealand; Canterbury Respiratory Research Group, 40 Stewart Street, Christchurch, New Zealand; Respiratory Services, Christchurch Hospital, Private Bag 4710, Christchurch, New Zealand; New Zealand Brain Research Institute, University of Otago at Christchurch, PO Box 4345, Christchurch, 8140 New Zealand; Oral Health Services, Christchurch Hospital, Private Bag 4710, Christchurch, New Zealand; Department of Medicine, University of Otago at Christchurch, PO Box 4345, Christchurch, 8140 New Zealand

**Keywords:** Very low birth weight, Infant, Population based, Follow-up, Psychosocial development, Young adult, Outcomes, Health related quality of life, Cardiovascular, Respiratory function tests

## Abstract

**Background:**

Very low birth weight (less than 1500 g) is associated with increased morbidity and costs of health care in childhood. Emerging evidence suggests these infants face a range of health and social problems as young adults. We studied all New Zealand very low birth weight infants born in 1986 (when 58 % were exposed to antenatal corticosteroids) in infancy, with later follow-up at 7 to 8 years and 23 to 24 years. We now aim to assess the cohort at 26–28 years compared with controls.

**Methods/design:**

The case sample will comprise a minimum of 250 members of the 1986 New Zealand national very low birth weight cohort (77 % of survivors). Outcomes will be compared with a control group of 100 young adults born at term in 1986. Following written informed consent, participants will travel to Christchurch for 2 days of assessments undertaken by experienced staff. Medical assessments include growth measures, vision, respiratory function, blood pressure and echocardiogram, renal function, dental examination and blood tests. Cognitive and neuropsychological functioning will be assessed with standard tests, and mental health and social functioning by participant interview. A telephone interview will be conducted with a parent or significant other person nominated by the respondent to gain a further perspective on the young person’s health and functioning. All those born at less than 28 weeks’ gestation, plus a random subset of the cohort to a total of 150 cases and 50 controls, will be offered cranial magnetic-resonance imaging. Statistical analysis will examine comparison with controls and long-term trajectories for the very low birth weight cohort.

**Discussion:**

The research will provide crucial New Zealand data on the young adult outcomes for very low birth weight infants and address gaps in the international literature, particularly regarding cardiovascular, respiratory, visual and neurocognitive outcomes. These data will inform future neonatal care, provide evidence-based guidelines for care of preterm graduates transitioning to adult care, and help shape health education and social policies for this high risk group.

**Trial registration:**

Australian New Zealand Clinical Trials Registry ACTRN12612000995875. Registered 1 October 2012

**Electronic supplementary material:**

The online version of this article (doi:10.1186/s12887-015-0413-9) contains supplementary material, which is available to authorized users.

## Background

Infants born very low birth weight (VLBW: <1500 g) or very preterm (VP: <32 weeks gestation) account for around 2 % of live births but between 50–75 % of the work load of neonatal intensive care units (NICUs) [1]. Whilst survival rates for these infants now exceed 90 %, [2, 3] what is most important for individuals, their families and society is the quality of that survival in the longer term. Knowledge of longer-term outcomes is also crucial to inform current neonatal care.

An extensive literature has documented increased rates of health problems amongst surviving VLBW/VP infants compared with full term infants in their early years, including sudden infant death syndrome, feeding problems, respiratory disorders, and increased hospitalisation [4]. Adverse outcomes are also common throughout childhood and include poor growth, neurosensory impairments such as cerebral palsy (CP) in up to10 % or more, and up to 50 % have behavioural problems, cognitive delay, or educational underachievement [5–11]. Woodward [12] reported that 72 % of VP infants have some white matter (WM) changes on cranial MRI scans at term equivalent, and that moderate or severe changes (in 21 %) were predictive of CP and neuro-cognitive problems in early childhood. These abnormalities include reduced WM volume, ventriculomegaly, thinning of the corpus callosum and delayed myelination. The impacts of these neonatal neurological abnormalities on adult brain structure and function are not known. However, findings from cross sectional studies in childhood and adolescence, [13–15] and a recent cohort study, [16] suggest that abnormalities persist and may be irreversible.

Data are also now emerging on a range of functional and health related challenges faced by former VLBW/VP infants as they reach young adulthood, [17, 18] although the majority appear to do well overall [19–21]. Scandinavian data show that preterm birth and particularly lower gestational age is associated with increased mortality in young adulthood, [22, 23] with congenital abnormalities, respiratory, endocrine and cardiovascular problems all increasing hazard ratios for risk of death [23]. Studies of growth of VLBW/VP children show that following early growth failure there is some catch-up that may continue through adolescence, although young adults remain shorter and often lighter than their peers [24]. Specific health issues found to be more prevalent include an increased risk of hypertension, [18, 25, 26] impaired insulin sensitivity, [27] glucose tolerance, [28] and renal function [29, 30] and abnormal respiratory function [25, 31]. VLBW children have a greater risk of asthma at school age than controls [32] and infants who had chronic lung disease have poorer lung function in late adolescence [25]. In addition, most reports to date indicate that former VLBW/VP young adults do have lower median IQ scores than their peers, even after excluding those with neurosensory impairments and controlling for confounding factors. Relatively fewer VLBW/VP survivors graduate from high school or enter tertiary education [17, 18, 33]. And even when free of major physical disability, VP young adults performed less well on tasks involving mental flexibility and response inhibition [34].

There are no New Zealand data in this crucial area and important gaps exist in the international literature. To address both these issues we will follow-up the 1986 New Zealand national cohort of VLBW infants, at age 26–28 years.

In 1986 we enrolled all 413 New Zealand VLBW infants admitted to a neonatal intensive care unit (NICU) as part of a prospective study of acute retinopathy of prematurity. Of these, 338 infants (82 %) survived to discharge home [35, 36]. Extensive perinatal data collection showed that at birth 58 % had received antenatal steroids, 132 (32 %) were <1000 g, 126 (31 %) were <28 weeks gestation, 103 (25 %) were small for gestational age, and 95 (23 %) were Maori. Survival for both those <1000 g and <28 weeks was 64 % [2, 36].

This cohort was followed-up at 7 to 8 years and assessments conducted to examine visual and developmental outcomes. By this age a further 12 children had died, 17 were traced to an overseas address, seven families declined follow-up and four were untraced. Hence we assessed 298 children (96 % survivors resident in NZ, 91 % all survivors): 5 % had severe disability, 5 % moderate disability and 15 % mild disability, which was principally an IQ between 1 to 2 SDs below the mean [9]. Compared with the Christchurch Health and Development Study cohort studied at the same age, [37] the VLBW cohort had higher rates of behavioural problems (conduct disorders, attention problems, anxiety/withdrawal), and poorer school achievement [10]. These differences increased with decreasing birthweight amongst the VLBW sample.

In 2009–10, when the members of the cohort were 23 to 24 years old, they were re-contacted as part of a feasibility study in preparation for funding applications for the current project. VLBW graduates were traced through their addresses at 7 to 8 years, grandparents’ addresses, general practitioners, National Health Index (NHI) codes (NHI – a unique person identifier used within the New Zealand health system since 1993), via the electoral rolls and, in a limited number of cases, by local advertisements. Checks were made with Statistics New Zealand to see if any individuals had died since last follow-up. A comparison group (controls) of individuals who were born full-term (FT) in New Zealand in 1986 and were not admitted for NICU care, was recruited either through peer nomination by cohort members or via random sampling from electoral rolls, ensuring balance with respect to the gender, ethnicity and regional distribution of the sample.

A total of 276 (85 % of 324) survivors were retraced and 230 (71 %) enrolled in this pilot (Phase 1) study, which comprised a face-to-face interview to collect information about each participant’s social background, health, education and social role outcomes, supplemented by information from medical records [38]. Sixty-nine controls were also recruited.

The present (Phase 2) study has two primary aims.

The first aim is to assess the health, mental status, functional outcome and quality of life in young adulthood of a NZ national VLBW cohort born in 1986 compared with a group of full-term children born in the same year.

We hypothesise that compared to their same age full term peers, adults born VLBW will, at age 26–28 years, have:

1. Lower levels of school attainment and tertiary educational participation, more limited occupational choice and higher levels of welfare dependence
2. Higher levels of inattention, impulsivity and emotional problems (i.e., symptoms of anxiety and depression), but lower levels of alcohol and drug use/abuse, antisocial behaviour and police contact.
3. Lower social participation and poorer social functioning
4. Poorer performance on measures of cognitive ability, information processing and executive functioning
5. Increased rates of cardiovascular, endocrine, pulmonary, renal and visual abnormalities and pathology
  *Specifically, regarding cardiovascular assessments, we anticipate:*
    Lower reactive hyperaemia consistent with more endothelial dysfunction
    Echocardiograms that show more abnormalities in LV size and function
    Elevation of BNP and other cardiac biomarkers and related signalling peptides, that reflect the degree of cardiovascular abnormality and will predict future cardiovascular events
  *regarding endocrine assessments, we anticipate:*
    Higher indices of insulin resistance and glucose intolerance
  *regarding pulmonary function, we anticipate:*
    Impaired pulmonary function, including reduced lung volumes and diffusing capacity, reduced exercise capacity, and higher rates of respiratory morbidity.
  *regarding renal function, we anticipate:*
    Higher microalbumin excretion and lower creatinine clearance
  *regarding vision, we anticipate:*
    Less favourable structural outcomes and impaired functional ability
6. Poorer dental health and dental hygiene
7. Increased rates of both macro- and micro-structural abnormalities on cranial MRI scans

The second aim is to assess whether childhood health and developmental outcomes are predictive of later young adult outcomes within the VLBW cohort.

We hypothesise that within the VLBW cohort:

8. Young adults who were characterised by significant disability/cognitive impairment at 7 to 8 years will fare less well on measures 1–4 than VLBW adults without early impairment [39].
9. Young adults who had retinopathy of prematurity (ROP) and visual impairments at 7 to 8 years will have more adverse visual problems than those who had no ROP.
10. Young adults who had neonatal chronic lung disease (CLD) will have more adverse respiratory function than those who no CLD
11. Increased duration of breastfeeding will be associated with a small but measurable advantage in cognitive outcomes
12. There will be increasing social and economic disadvantage with decreasing birthweight and increasing prematurity.

In our preliminary (Phase 1) study we re-established the cohort and began to address hypotheses 1–3. This Phase 2 study will extend the research to focus on health issues and neurocognitive outcomes but also to update the information collected as part of Phase 1.

## Design and methods

### Study population

The case sample will comprise a minimum of 250 members of the 1986 NZ National VLBW cohort (77 % of survivors). In Phase 1 the proportion (%) of the 230 survivors seen and other survivors not assessed (94) who were <1000 g was 25.2 and 27.1; were <28 weeks is 24.8 and 24.0; are male is 47.0 and 52.1; are Maori is 26.1 and 22.1; had moderate/severe disability at 7 to 8 years is 10.3 and 6.7 – all being not significantly different. Outcomes for the VLBW cohort will be assessed against a comparison sample of 100 same age term controls recruited via a process of peer nomination and random sampling from electoral rolls, ensuring balance with respect to the gender, ethnicity and regional distribution of the sample.

### Procedures

As part of Phase 1 all participants have been informed about Phase 2 of the study and have consented to being approached to participate in these assessments. The study has also obtained comprehensive contact information for each participant, plus a number of individuals not studied in Phase I, and we do not anticipate major difficulties in tracing and consenting participants for Phase 2. Following consent, participants will be brought to Christchurch for a 2 day multidisciplinary evaluation (an average of three participants a week). Provision will be made for a support person to travel with the participant if this is required. All costs of travel, accommodation and food will be met by the study. For the small number of the cohort members who are too compromised to travel to Christchurch due to disability severity and/or care needs we will seek information by means of a questionnaire and, where possible, a visit from one of the research team (Additional files 1, 2, 3, 4, 5, 6).

All research procedures will be conducted in existing facilities in the University of Otago, Christchurch or Canterbury District Health Board (CDHB). Participants will be scheduled to arrive the night before, have fasting blood tests the next morning and a series of assessments over 2 days. Study personnel will meet each participant and assist their navigation between laboratory sessions. To avoid problems of fatigue, participants will have the opportunity to take breaks as necessary and refreshments as required. Transportation will also be provided when needed.

Medical investigations will be undertaken in the University of Otago, Christchurch, Department of Medicine Nicholls Clinical Research Centre and the CDHB Respiratory Physiology Laboratory facilities.

Assessments will be conducted by appropriately trained, medically qualified staff, who will be blinded to the participant’s background (except where noted otherwise), and will include:

- ▪ Standardised questionnaires on health, including respiratory [40] and bowel problems.
- ▪ Growth-height (Harpenden stadiometer), weight, BMI (Weight/Height^2^), waist-hip ratio, head circumference.
- ▪ Total body fat estimated by bioelectrical impedance analysis (BIA).
- ▪ Visual assessment. Glasses prescription-measured in focimeter. Distance visual acuity using standardised ETDRS chart (with glasses if worn). Contrast sensitivity testing with Pelli-Robson charts (which reflects *functional* visual ability). Autorefraction. Retinal photographs to digitally record macula, optic disk, paracentral and peripheral retina.
- ▪ Blood pressure assessed using a random zero machine (seated, after rest for 5 min, cuff size 2/3 upper arm length: mean of three readings)
- ▪ Echocardiogram for left ventricular volume, mass and function, valves, ascending aorta dilatation. Measurement of myocardial velocities and deformation.
- ▪ Endothelial dysfunction assessment by peripheral arterial tonometry (EndoPAT), [41] consisting of digital recording equipment and two finger thimble-like probes.
- ▪ Respiratory Function Tests. Forced expiratory volume in 1 s (FEV_1_), Forced Vital Capacity (FVC) and FEV_1_/FVC [42]. Static lung volumes [43]. Diffusing capacity of CO [44]. Single breath nitrogen washout [45]. Maximal exercise testing [46].
- ▪ Renal function, assessed by a) creatinine clearance (C_cl_) – [Blood test for plasma creatinine in mmol/L. C_cl_ calculated from Cockcroft-Gault approximation] and b) early morning urine for albumin-creatinine ratio [mg/mmol: <2.2 normal; 2.2–22.6 microalbuminuria; >22.6 proteinuria].
- ▪ Additional venous blood sample for:

Fasting insulin and glucose – (insulin sensitivity calculated from QUICKI [47]) lipids; glycated haemoglobin; cotinine.

Plasma hormones including B-Type Natriuretic Peptide (BNP), amino terminal proBNP (NT-proBNP), C-Type Natriuretic Peptide (CNP), amino terminal proCNP (NT-proCNP), high sensitivity troponin T (hsTNT), renin, aldosterone.

Stored plasma sample for future use – investigation of gene-environment interactions; biochemical assays.

Blood volume: 5 × 9 ml EDTA, plus 3 ml A2, plus 2 × 9 ml lithium heparin for hormones and storage. Plus 4 ml lithium heparin for biochemistry. Total 70 ml.

Total time for tests including breaks is estimated at about 2.5–3.5 h (medical/cardiology 1–1.5 h, respiratory tests 1–1.5 h, visual assessments 0.5 h).

Oral Health investigations will be undertaken by dentists from the oral health service of the Canterbury District Health Board using a standardised protocol from the 2009 New Zealand Oral Health Survey [48]. The examination will include assessments of the wearing of dentures; history of orthodontic treatment; the health of oral mucosa; tooth loss, presence of decay, and dental treatments; plaque, calculus, gingivitis; periodontal tissue destruction; decay experience of coronal and root surfaces; and the presence of enamel defects. Total examination time will be approximately 45 min. Dental examination will be supplemented by questionnaire data on current problems or concerns about oral health, practise of dental hygiene and use of dental health services.

Neuropsychological Functioning will be assessed in other existing facilities in the University of Otago, Christchurch, by research staff with appropriate training and qualifications in psychometric test administration. The test room will be suitably quiet. The tester will be blinded to the participants’ background. Testing will take between 2 to 3 h for each participant, including breaks as needed, and will include assessments of:

- ▪ Global cognitive ability/IQ will be assessed using the *Wechsler Abbreviated Scale of Intelligence® – Second edition* (WASI-II), [49] an individually administered assessment of intelligence for people aged 6–90 years. This short form consists of four subtests including Block Design and Matrix Reasoning (which compose the Perceptual Reasoning Index) and the Vocabulary and Similarities subtests (which compose the Verbal Comprehension Index).
- ▪ Attention will be measured using the *Test of Everyday Attention* (TEA), [50] which assesses selective attention, sustained attention and switching attention.
- ▪ Working memory – Visual working memory will be assessed using a computer administered task adapted from the *Sternberg Spatial Working Memory Paradigm* [51]. Auditory working memory will be assessed using the two elevator counting tasks of the Test of Everyday Attention: *Elevator counting with distraction; Elevator counting with reversal.*
- ▪ Cognitive flexibility will be assessed by the *Comprehensive Trail Making Test* (CTMT), [52] comprising a standardized set of five visual search and sequencing tasks, and the *Brixton Spatial Anticipation Test*, [53] which is a measure for assessing the ability to detect and follow a rule.
- ▪ Visuo-spatial processing and planning will be assessed using the *Benton Judgement of Line Orientation – Form V* [54].
- ▪ Information processing speed will be assessed using the *Symbol Digit Modalities Test* [55]*.*

For participants with a physical or sensorineural disability some tests may not be possible, and where possible an alternative form of the test will be used.

#### Health and social functioning

Trained survey interviewers will update information from study Phase 1 on education, occupational status, health history and self-assessment of health status, quality of life measures, current mental health, substance use, family, peer and partner relationships and other aspects of social functioning. Interviews (2 to 3 h) will be conducted in private.

#### Parent/significant other report

To gain a further perspective on the young person’s health and functioning, a telephone interview will be conducted with a parent or significant other person nominated by the respondent. These interviews will gather a third person perspective on aspects of the young person’s health and health history, education and transition to adult roles, the young person’s behaviour and personality, social and partner relationships and related issues. Interviews will take 35–45 min and will be conducted by trained survey interviewers who are independent of the survey team interviewing the young person, but are not blinded their background.

#### Magnetic resonance imaging

We will undertake a cranial MRI scan on all participants who were born at <28 weeks gestation and a random sample of the VLBW cohort to bring the total having MRI scans to 150, plus a random 50 controls. Images will be acquired using a 3-Tesla GE scanner on the Christchurch campus and analyses undertaken by experienced staff blinded to the participants’ background. Scanning will take a total of 35–45 min comprising:

- ▪ Clinical data – T2 and T2 FLAIR images as part of standard clinical protocol for purposes of a radiologist read/referral/exclusion and qualitative scoring
- ▪ T1-weighted images will be used to assess brain growth and tissue distribution
- ▪ Diffusion Tensor imaging (DTI) will assess white matter microstructural development (fractional anisotropy).
- ▪ Arterial Spin Labelling (ASL) – a non-invasive functional measure of cerebral blood flow that produces quantification of cerebral perfusion and has good concordance with ^15^O-PET [56]
- ▪ Resting State Functional Connectivity (fcMRI) which provides a method to evaluate regional interactions in the brain.

Both ASL and fcMRI may be clinically useful as biomarkers for individuals at increased risk of later cognitive impairment. This protocol, with the addition of ASL, is the same as that being used at 12 years of age in a cohort of former <32 week gestation children, who also had MRI scans at term equivalent, [12] and this may allow early clues as to longitudinal trajectories of brain injury and neural plasticity.

### Additional studies

Although not part of the primary protocol for the New Zealand 1986 VLBW Follow-up Study, we will also undertake two additional studies with separate funding.

1. DNA methylation and outcomes in VLBW young adults. We will investigate whether young adults born with VLBW have differences in DNA methylation at specific sites in the genome (in peripheral blood samples), compared with a matched normal birthweight group, and whether these traits are associated with growth and metabolic health outcomes as young adults. Separate consent is required for this study, which also requires an additional 10 ml blood sample be taken.
2. C type Natriuretic Peptide (CNP) and vascular risk in VLBW young adults. CNP is a paracrine vasoprotective peptide secreted by the vascular endothelium, and which has anti-inflammatory actions in rodents and has been shown to be atheroprotective. Very little CNP normally enters the circulation but a stable product of the prohormone (amino terminal proCNP, NTproCNP) can be readily measured in plasma and serves as a marker of CNP production in tissues. Recent studies by the Christchurch group have defined the normal age and gender related ranges of plasma NTproCNP concentration in 250 healthy adult subjects without known history of cardiovascular disorder [57]. We will compare the plasma NTproCNP concentrations in VLBW young adults and term born controls and investigate their relationship with individual vascular risk factors and with adult height.

### Statistical analysis and statistical power

The collected data will enable two types of analyses to be conducted. Between group comparisons: The principal analyses will examine between group differences in outcome (health, neurocognitive assessments, social functioning), using standard statistical analyses: chi square for differences in proportions; *t*-test for independent samples or analysis of variance for differences in means of continuous outcomes; Poisson or negative binomial regression for differences in rates. Where outcome data are non-normally distributed appropriate data transformations (eg logarithmic) may be conducted. The VLBW group may be further subdivided to test for differential effects by severity of disability at age 7 to 8, or by birthweight or gestation. Comparisons may be further adjusted if necessary for between group differences in family background characteristics using regression methods based on the generalised linear model [58]. Assuming a minimum of 250 recruits from the VLBW cohort and 100 controls, the study will have 80 % power at alpha = .05 to detect differences between groups of 30 SD or greater on continuous outcomes, and ORs in the region of 2.0–3.5 for dichotomous outcomes (depending on the base rate) [59]. This suggests the study will have adequate power to detect effect sizes in the small to moderate range. These differences are well within the anticipated effect sizes based on existing reports [17, 18]. Subdividing the VLBW group (for example by level of prior disability) is unlikely to compromise study power since the effect sizes are likely to increase for more extreme groups.

Outcome trajectories amongst the VLBW cohort: For a number of outcomes, for example visual, physical growth, it will be possible to examine across time continuities and/or trajectories in outcome from childhood to adulthood within the VLBW cohort. Depending on the outcome under consideration, analysis methods may range from simple tabular methods documenting outcome in young adulthood conditional on childhood status, to simple regression models predicting outcome in adulthood from measures in childhood, to more complex methods for repeated measures data based on a generalised estimating equation approach [60] to model changes in outcome trajectory.

## Ethics

The study has received ethics approval from the Upper South B Regional Ethics Committee (superseded by the Southern Health and Disability Committee) (ref: URB/12/05/015).

## Discussion

The study of adult outcomes after VLBW/VP birth is a relatively new field and one which is expanding, but there are important gaps in current knowledge. Many cohorts date from the 1970’s or early 1980’s when few infants received antenatal steroids, which reduce both mortality and morbidity [61]. Some studies are from single institutions, [17, 62] or a group of institutions, including the Helsinki Study of Very Low Birth Weight Adults based on 166 VLBW infants born in 1978–85 and which has been highly productive [26, 63]. Of the few population based studies most are regional [17, 18] or rely on constrained national registry data [19] (Swedish, [64, 65] Norwegian [66] and Danish [67] studies). The Netherlands study of VP/VLBW infants born in 1983 [68, 69] is most similar to our NZ cohort but these infants were not exposed to antenatal corticosteroids. As more data become available, understanding trends across studies as well as differences between them will become important. The New Zealand 1986 VLBW Follow-up Study investigators are committed to collaborating with other studies as part of the Adult Born Preterm International Collaboration (APIC), which has been initiated by the Helsinki, Finland group [70].

## Additional files

Additional file 1:
**NZ 1986 VLBW FU Study Cohort Information Sheet May 2013 Final.**
Additional file 2:
**NZ 1986 VLBW FU Study Controls Information Sheet May 2013 Final.**
Additional file 3:
**NZ 1986 VLBW FU Study Information Sheet Genetics August 2012.**
Additional file 4:
**NZ 1986 VLBW FU Study Consent Form Genetics Jan 2013.**
Additional file 5:
**NZ 1986 VLBW FU Study Consent Form Genetics Jan 2013.**
Additional file 6:
**NZ 1986 VLBW FU Study Explanation of procedures June 2013.**

## Abbreviations

VLBW: Very low birth weight
VP: Very preterm
NICU: Neonatal intensive care unit
CP: Cerebral palsy
WM: White matter
MRI: Magnetic resonance imaging
IQ: Intelligence quotient
SD: Standard deviation
NHI: National Health Index
FT: Full-term
NZ: New Zealand
ROP: Retinopathy of prematurity
CLD: Chronic lung disease
CDHB: Canterbury District Health Board
BMI: Body-mass index
BIA: Bioelectrical impedance analysis
ETDRS: Early treatment diabetic retinal study
EndoPAT: Peripheral arterial tonometry
FEV_1_: Forced expiratory volume in 1 s
FVC: Forced vital capacity
CO: Carbon monoxide
C_cl_: Creatinine clearance
QUICKI: Quantitative insulin sensitivity check index
BNP: B-Type natriuretic peptide
CNP: C-Type natriuretic peptide
hsTNT: High sensitivity troponin T (hsTNT)
EDTA: Ethylenediaminetetraacetic acid
WASI: Wechsler Abbreviated Scale of Intelligence®
TEA: Test of everyday attention
CTMT: Comprehensive trail making test
DTI: Diffusion tensor imaging
ASL: Arterial spin labelling (ASL)
fc: Functional connectivity
APIC: Adult born preterm international collaboration
