# Supplementary material for: The New Zealand 1986 very low birth weight cohort as young adults: mapping the road ahead
Source: BMC Pediatr. 2015 Aug 5;15:90. doi: 10.1186/s12887-015-0413-9 (PMC4526306; doi:10.1186/s12887-015-0413-9)
Supplement: Additional file 2: — NZ 1986 VLBW FU Study Controls Information Sheet May 2013 Final. [file 12887_2015_413_MOESM2_ESM.pdf]

UNIVERSITY of OTAGO

Te Whare Wananga o Otago

The New Zealand 1986 VLBW cohort as young adults: mapping the road ahead
INFORMATION SHEET for CONTROLS

Principal Investigator: Professor Brian Darlow, Department of Paediatrics, University of Otago, Christchurch, PO Box 4345, Christchurch. Ph: (03) 364-0747

Background:
You are invited to take part in this study looking at health and developmental outcomes in young adulthood for New Zealand children born in 1986 who were born very early (premature) or of very low birth-weight (<1500 grams). Since the 1980s changes in medical technology have resulted in dramatic improvements in the survival of children born very early or very small. However, there have been ongoing concerns that premature infants have increased risks of some health problems and higher rates of disability and developmental problems than other children. Currently, around the world there is considerable interest in the long term outcomes for these infants, as substantial numbers of premature and low birthweight infants are now growing into adulthood. The purpose of this study is to obtain information on the long term outcomes of prematurity or very low birthweight for New Zealand children.

This study involves comparing health and developmental and life course outcomes around age 26 for two groups of young adults: (a) the first group will comprise all those survivors of the group of children born in New Zealand in 1986 and weighing <1500 grammes at birth; (b) the second group will comprise a comparison series of young adults who were also born in New Zealand in 1986 but who were not born premature or of very low birthweight. In this way it will be possible to examine the extent to which individuals born very early may be disadvantaged compared to the wider population in terms of their health, adjustment and life functioning outcomes in young adulthood.

Most in the control group will have already taken part in the study at 23-24 years of age, when you were invited to undertake an interview with a research nurse on matters relating to your circumstances and life experiences. If you are being contacted for the first time this is because your name has been identified from the electoral rolls as a possible candidate for enrolment in the study, and we would like to invite you to consider participating in this research, providing you were born in New Zealand in 1986, and you were not born early (premature) or of very low birthweight. Our Research Nurse will discuss these issues with you when she contacts you about the study.

Whether you have taken part in the study previously or not, we are now contacting you to invite you to participate in this study looking at health issues and comprising a number of medical investigations and tests of ability and functioning.

What will the Study Involve?
The study will involve a trip to Christchurch for a two day series of investigations. We will pay for your travel to Christchurch and your accommodation and will provide meals. There are several parts to the investigation.

1. Medical investigations, including:

- Measurement of height, weight, waist and hip circumferences
- Estimation of body fat by a simple bed-side test called bioelectrical impedance – the test is quite painless and measures flow of a tiny current between your wrist and ankle

- Assessment of vision
- Blood pressure
- A measure of blood flow in a finger called "arterial tonometry" – your index finger wears a thimble-like probe and a blood pressure cuff on your arm is inflated and deflated several times
- An echocardiogram, which uses ultrasound to take pictures of your heart. This procedure is painless and does not require any preparation or injections. The examination takes about 25 minutes and there are no known side effects
- Respiratory (breathing) function tests, which will include a simple exercise test
- Blood tests taken from a vein (total 70 ml blood)
- We will also ask you to provide an early morning urine sample.
- We will ask some standard questions relating to health, respiratory and bowel problems
- Dental examination – questionnaire; visual inspection and gentle dental probe of gums

The total time for these tests, including breaks, will be 3 to 4 hours (visual assessment ½ hour, respiratory tests 1 to 1½ hours, cardiac/other 1 to 1½ hours, dental ½ to 1 hour)

2. Tests of ability and functioning, including:

- An IQ test using the Wechsler Adult Intelligence Scales
- Tests of memory
- Tests of "executive function" which is a set of mental processes that help us organise thoughts and actions
- Tests of ability to maintain focussed attention on an activity
- Tests of visuo-spatial processing
- Tests of information processing speed

Most of these tests comprise a sort of "game". Including breaks between tests the total time for testing will be 2 to 3 hours

3. An interview about aspects of your current life and circumstances:

This will be similar to the interview we conducted with you previously at age 23-24. The aim is to gather up to date information on a range of topics including your education, employment, health, adjustment, social relationships and related matters.

The interview will take about an hour

4. In addition some participants will be invited to have **a cranial (head) magnetic resonance image (MRI) scan:**

If you agree to this you will lie on a table which slides into a tunnel-shaped scanner. The scanner uses powerful magnets and radio waves to create pictures of the brain. No radiation is involved and the procedure is painless. You will hear a variety of loud noises but we will provide you with earplugs to compensate for this. Metal objects are not allowed in the room and you will be asked about these. The Christchurch Radiology Group MRI information sheet is attached.

The test takes 35 to 45 minutes

With your consent we would also like to talk to a parent (or someone else close to you) to gain another perspective on some of the things we have discussed with you.

Our goals in collecting this information are to obtain an accurate and realistic assessment of the health issues, problems and functioning difficulties facing young New Zealanders who were born

premature or of very low birthweight as they mature into adulthood, and to better predict any future health problems. In appreciation of your help with this research and to compensate you for any inconvenience or loss of earnings, you will be reimbursed $200 in total for the two days in exchange for your participation.

Participation

Your participation in the study is entirely voluntary (your choice). You do not have to take part in this study, and if you choose not to take part we will respect your choice.

You are free to withdraw from the study at any time, for any reason. You do not have to undertake all the tests or answer all the questions. You may discontinue participation in any part of the assessment, or the rest of the study, at any time.

If you have any questions or concerns about your rights as a participant in this research study you can contact an independent health and disability advocate. This is a free service provided under the Health and Disability Commissioner Act. Telephone: (NZ wide) 0800 555 050. Free Fax (NZ wide): 0800 2787 7678 (0800 2 SUPPORT). Email (NZ wide): advocacy@hdc.org.nz

Risks and Safety

The main risk might be tiredness from doing assessments – we will give opportunities for breaks, refreshments, etc as required. Blood tests sometimes result in minor discomfort and/or bruising. The MRI scan (if included in your assessment) is noisy and no metal objects are allowed in the room.

In the unlikely event of a physical injury as a result of your participation in this study, you may be covered by the ACC under the Injury Prevention Rehabilitation and Compensation Act. ACC cover is not automatic and your case will need to be assessed by the ACC according to the provisions of the 2002 Injury Prevention Rehabilitation Act. If your claim is accepted by the ACC, you might still not get any compensation. This depends on a number of factors such as whether you are an earner or non-earner. ACC usually provides only partial reimbursement of costs and expenses and there may be no lump sum compensation payable. There is no cover for mental injury unless it is a result of physical injury. If you have ACC cover, generally this will affect your right to sue the investigators. If you have any questions about ACC, contact your nearest ACC office or the investigator.

You are also advised to check whether participation in this study would affect any indemnity cover you have or are considering, such as medical insurance, life insurance and superannuation.

Some of the blood samples collected for the study will be stored for possible future use. At the end of the study any remaining samples will be destroyed using standard disposal methods. You have the option of requesting disposal with karakia if you wish. Some blood sample analysis may be performed overseas by collaborating investigators. Any blood samples sent to sites outside New Zealand will be labelled with a coded number only. Unused samples will be destroyed by standard disposal (disposal by karakia will not be available in this instance).

What health records will be accessed?

With your consent, your GP will be informed about your participation in this study and, again with your consent, we will access info on your medical history from your GP. Information may also be obtained from your medical records and through the New Zealand Health Information Service, a division of the Ministry of Health, for admissions or hospital attendances outside Christchurch

Confidentiality:

We fully appreciate that the information you provide is personal and we would advise you that all

material that you provide us will be treated in the utmost confidence. Your identity will not be revealed in any reports based on this study. Other research groups may be supplied with data from the NZ very preterm young adults study but in all cases this information will be anonymous.

The study has a comprehensive security system which ensures that all information you provide and from your tests is stored in anonymous form on computer files and that no data that can be linked to an individual can be accessed without knowledge of this security system. This knowledge is confined to members of the research team.

Who is funding the study?
The Health Research Council of New Zealand and the Child Health Research Foundation.

Help and Assistance:
If you have any questions or there is something you would like to discuss please do not hesitate to contact the study director, Dr Brian Darlow, or our Project Manager (contact details below). If you are telephoning from outside Christchurch you may ring collect and we will pay the charges.

Feedback and Results:
We are happy to provide you with feedback on the findings of the study. There is likely to be some time lag between when your assessment is conducted and when the findings of the study finally become available. However, if at any stage you feel you would like an update on the research or have any questions about the study, please do not hesitate to contact us.

Should we identify any clinically significant abnormal results, we undertake, with your permission, to make these known to your GP, who can then explain them to you.

We are committed to treating all our study participants in a fair and ethical manner. The study has received ethical approval from the Southern Health and Disability Ethics Committee (Ethics ref URB/12/05/015).

Finally, we would like to thank you for giving up your time to assist us with this research.

Investigators:

Brian Darlow	Department of Paediatrics, University of Otago, Christchurch<br>Ph (03) 364-0747 or (03) 364 4699 and ask to page 5038<br>e-mail: brian.darlow@otago.ac.nz
John Horwood	Department of Psychological Medicine, University of Otago, Christchurch. Ph (03) 372-0406
Lianne Woodward	Department of Psychology, Washington University, St Louis, USA
John Elliott	Department of Medicine, Cardioendocrine Research Group<br>Ph (03) 364-0640 and ask to page 8222
Richard Troughton	Department of Medicine, Cardioendocrine Research Group<br>Ph (03) 364-0826

Project Manager:

Julia Martin	Department of Paediatrics, University of Otago, Christchurch.<br>Ph: (03) 378 6437 Mobile: 021 248 7999<br>e-mail: julia.martin@otago.ac.nz
