# Supplementary material for: The New Zealand 1986 very low birth weight cohort as young adults: mapping the road ahead
Source: BMC Pediatr. 2015 Aug 5;15:90. doi: 10.1186/s12887-015-0413-9 (PMC4526306; doi:10.1186/s12887-015-0413-9)
Supplement: Additional file 3: — NZ 1986 VLBW FU Study Information Sheet Genetics August 2012. [file 12887_2015_413_MOESM3_ESM.pdf]

UNIVERSITY of OTAGO

Te Whare Wananga o Otago

The New Zealand 1986 VLBW cohort as young adults: mapping the road ahead

Consent Form

1. I have been invited to take part in this study looking at health outcomes in young adulthood for very preterm babies born in New Zealand in 1986 compared to same age controls, who were born at full term.

2. I have read and understand the Information Sheet dated 17 May 2013 for volunteers taking part in the study. I have had the opportunity to discuss the study. I am satisfied with the answers I have been given.

3. I have had the opportunity to use whanau support or a friend to help me ask questions and understand the study.

4. I understand that:

   - My taking part in this study is voluntary (my choice).
   - I am free to withdraw from the study at any time and for any reason.
   - I am free to discontinue any tests at any time, and to refuse to answer any questions I don't want to answer.
   - My participation in this study is confidential, and no information that could identify me will be used in any reports on this study.

5. I have had time to consider whether to take part.

6. I know whom to contact if I have any questions about the study.

7. I understand that this study has received ethical approval from the Southern Health and Disability Ethics Committee.

8. I understand that the study: (please TICK boxes)

   ☐ Will involve completing a questionnaire into medical history

   ☐ Will involve providing a blood sample

   ☐ Will involve an ultrasound examination of the heart (echocardiogram)

   ☐ Will include respiratory function tests including an exercise test

   ☐ Will involve tests of vision

   ☐ Will include an IQ test

   ☐ Will include tests of memory, executive functioning, attention and mental processing

   ☐ Will include a dental examination – visual inspection and gentle probe of gums

☐ May include a cranial (head) magnetic resonance imaging (MRI) scan

The nature of these tests and the purposes for which they are being undertaken have been explained to me and I understand that all information obtained will be treated in the strictest confidence and will not be released to any third party in a form that could be identified with me.	☐ YES ☐ NO

9. Medical records: I agree to members of the research team having access to the following sources of information concerning myself:

Information on my health history as recorded in my General Practitioner, specialist or other health practitioner records	☐ YES ☐ NO
Information on my health history as recorded in hospital or New Zealand Health Information Service records	☐ YES ☐ NO

10. Blood samples:

1 I consent to the researchers storing a specimen of my blood for its later use as part of this study or other research approved by a Health and Disability Ethics Committee	☐ YES ☐ NO
I consent for some of the stored blood taken from me during the study to be sent overseas to be tested for new medical markers if it is not possible to do this in New Zealand	☐ YES ☐ NO

I request any remaining blood samples be disposed of at the end of the study to (please tick one):

☐ Using standard disposal methods only

☐ Using standard disposal methods with appropriate karakia

11.	I consent to the use of my data for future related studies, which have been given ethical approval from a Health and Disability Ethics Committee.	☐ YES ☐ NO
12.	I consent to results of the testing being given to my GP	☐ YES ☐ NO
13.	I wish to receive a summary of the results of the study. (There may be a considerable delay between collection of the data and when the results become available).	☐ YES ☐ NO

14. I hereby consent to take part in this study.

Signature:__________________________________ **Date:** d____/m______/y______

Full Name: (please print): ____

Investigators:

Brian Darlow: Department Paediatrics, University of Otago, Christchurch.

John Horwood: Department of Psychological Medicine, University of Otago, Christchurch

Lianne Woodward: Department of Psychology, Washington University, St Louis, USA (Formerly University of Canterbury)

John Elliott: Department of Medicine, Cardioendocrine Research Group, University of Otago, Christchurch

Richard Troughton: Department of Medicine, Cardioendocrine Research Group, University of Otago, Christchurch

Contact phone number: Brian Darlow: (03) 364-0747
Project Manager: Julia Martin (03) 378 6437 or 021 248 7999

Project explained by:

Signature: __________________________Date: ____________________________

Name: _____________________________ Role: ____________________________

General Practitioner Details:

GP Name: ____

Practice: ____

Address: ____

____
