# Supplementary material for: The New Zealand 1986 very low birth weight cohort as young adults: mapping the road ahead
Source: BMC Pediatr. 2015 Aug 5;15:90. doi: 10.1186/s12887-015-0413-9 (PMC4526306; doi:10.1186/s12887-015-0413-9)
Supplement: Additional file 4: — NZ 1986 VLBW FU Study Consent Form Genetics Jan 2013. [file 12887_2015_413_MOESM4_ESM.pdf]

UNIVERSITY of OTAGO

Te Whare Wananga o Otago

The New Zealand 1986 VLBW cohort as young adults: mapping the road ahead
INFORMATION SHEET for Genetic Profiles of Heart Disease

Principal Investigator: Professor Brian Darlow, Department of Paediatrics, University of Otago, Christchurch, PO Box 4345, Christchurch. Ph: (03) 364-0747

Background:

You have been invited to take part in a study looking at health and developmental outcomes in young adulthood for New Zealand children born in 1986 who were born very early (premature) or of very low birth-weight (<1500 grams), either as one or the group born very early or as one of the control group who were born at term.

As an addition to that study we are interested in trying to identify genes that might be associated with heart disease.

What is heart disease?

- Cardiovascular disease (diseases of the heart and blood vessels) is the leading cause of death in New Zealand, accounting for approximately 40% of all deaths per year. This compares with 26% for cancer. The commonest forms of cardiovascular disease include: angina, heart attack (myocardial infarction) and heart failure.
- The death rate from cardiovascular disease is more than twice as high in men as in women.
- Overall, 84% of deaths related to cardiovascular disease occur in people over 65 years of age.

How can I help?

We are researching the genes that may be associated with cardiovascular disease, by comparing the pattern of genes from people without heart disease with patients who have heart disease. We invite you to donate a blood sample so that we can extract DNA, which comprises the body's genetic code. You will also be asked to answer a short questionnaire detailing your relevant personal medical history and any family history of heart disease or related conditions.

Will the results of my genetic analysis remain confidential?

Your information and blood sample will be given a code number, so that laboratory personnel will not know your identity. The details of your medical history relevant to the study may be reviewed by a study investigator, but they will treat this information confidentially. Your DNA sample will be stored in secure laboratory facilities identified by your sample's unique code number.

The DNA information will NOT be used to identify individuals for any forensic or clinical purposes. The DNA will NOT be used to test for any known disease and no "test result" will be generated. Genetic data will be analysed in group comparisons only and is for confidential research use only and no feedback can be given to individual study members or their GP about their genes.

You may withdraw your sample from the study without explanation at any time and your sample and data will be destroyed.

What will the Study Involve?

If you agree to taking part in this addition to the main "NZ very preterm young adults study" it will mean an extra 10 ml of blood will be taken at the same time we take blood from a vein for other standard blood tests.

Participation:
Your participation in the study is entirely voluntary (your choice). You do not have to take part in this study, and if you choose not to take part we will respect your choice.

You are free to withdraw from the study at any time, for any reason.

If you have any questions or concerns about your rights as a participant in this research study you can contact an independent health and disability advocate. This is a free service provided under the Health and Disability Commissioner Act. Telephone: (NZ wide) 0800 555 050. Free Fax (NZ wide): 0800 2787 7678 (0800 2 SUPPORT). Email (NZ wide): advocacy@hdc.org.nz

Compensation
In the unlikely event of a physical injury as a result of your participation in this study, you may be covered by the ACC under the Injury Prevention Rehabilitation and Compensation Act. ACC cover is not automatic and your case will need to be assessed by the ACC according to the provisions of the 2002 Injury Prevention Rehabilitation Act. If your claim is accepted by the ACC, you might still not get any compensation. This depends on a number of factors such as whether you are an earner or non-earner. ACC usually provides only partial reimbursement of costs and expenses and there may be no lump sum compensation payable. There is no cover for mental injury unless it is a result of physical injury. If you have ACC cover, generally this will affect your right to sue the investigators. If you have any questions about ACC, contact your nearest ACC office or the investigator.

Help and Assistance:
If you have any questions or there is something you would like to discuss please do not hesitate to contact the study director Brian Darlow or our Project Manager (contact details below). If you are telephoning from outside Christchurch you may ring collect and we will pay the charges.

We are committed to treating all our study participants in a fair and ethical manner. The study has received ethical approval from the Regional Ethics Committee.

Investigators:

Brian Darlow	Department of Paediatrics, University of Otago, Christchurch<br>Ph (03) 364-0747 or (03) 364 4699 and ask to page 5038<br>e-mail: brian.darlow@otago.ac.nz
John Elliott	Department of Medicine, Cardioendocrine Research Group<br>Ph (03) 364-0640 and ask to page 8222
Richard Troughton	Department of Medicine, Cardioendocrine Research Group<br>Ph (03) 364-0826

Project Manager:

Name	Department of Paediatrics, University of Otago, Christchurch.<br>Ph<br>e-mail:
