# Supplementary material for: The New Zealand 1986 very low birth weight cohort as young adults: mapping the road ahead
Source: BMC Pediatr. 2015 Aug 5;15:90. doi: 10.1186/s12887-015-0413-9 (PMC4526306; doi:10.1186/s12887-015-0413-9)
Supplement: Additional file 5: — NZ 1986 VLBW FU Study Consent Form Genetics Jan 2013. [file 12887_2015_413_MOESM5_ESM.pdf]

UNIVERSITY of OTAGO

Te Whare Wananga o Otago

The New Zealand 1986 VLBW cohort as young adults: mapping the road ahead

Consent Form for Genetic Profiles of Heart Disease

I have been invited to take part in this study to provide a blood sample that will be processed for DNA studies for gene variants associated with risk of heart disease. I understand this is an optional addition to the main study looking at health outcomes in young adulthood for very preterm babies born in New Zealand in 1986 compared to same age controls, who were born at full term.

I have read and understand the Information Sheet dated 23 August 2012 for volunteers taking part in the study. I have had the opportunity to discuss the study. I am satisfied with the answers I have been given.

I have had the opportunity to use whanau support or a friend to help me ask questions and understand the study.

- my taking part in this study is voluntary (my choice).
- I am free to withdraw from the study at any time and for any reason.
- I am free to withdraw my blood sample at any time and have this sample destroyed.
- my participation in this study is confidential, and no information that could identify me will be used in any reports on this study.
- I have had time to consider whether to take part.
- I know whom to contact if I have any questions about the study.
- I understand that this study has received ethical approval from the Regional Ethics Committee.

I understand that the study will involve: (please TICK box)

☐ Providing a blood sample

The nature of these tests and the purposes for which they are being undertaken have been explained to me and I understand that all information obtained will be treated in the strictest confidence and will not be released to any third party without my written permission. ☐ YES ☐ NO

I wish to receive a summary of the results of the study ☐ YES ☐ NO

I hereby consent to take part in this study.

Signature:_________________________________ **Date:** d____/m______/y______

Full Name: (please print):

____

Investigators:

Professor Brian Darlow:	Dept Paediatrics, Christchurch School of Medicine, University of Otago, Christchurch.
John Elliott	Dept Medicine, Cardioendocrine Research Group, U Otago
Richard Troughton	Dept Medicine, Cardioendocrine Research Group, U Otago

Contact phone number: Brian Darlow: (03) 3640-747
Project Manager: *To follow, when appointed*

Project explained by:

Signature: ________________________**Date:** __________________________

Name: ___________________________ **Role:** __________________________
