# Supplementary material for: The New Zealand 1986 very low birth weight cohort as young adults: mapping the road ahead
Source: BMC Pediatr. 2015 Aug 5;15:90. doi: 10.1186/s12887-015-0413-9 (PMC4526306; doi:10.1186/s12887-015-0413-9)
Supplement: Additional file 6: — NZ 1986 VLBW FU Study Explanation of procedures June 2013. [file 12887_2015_413_MOESM6_ESM.pdf]

# NEW ZEALAND VERY LOW BIRTHWEIGHT YOUNG ADULTS:

# MAPPING THE ROAD AHEAD

# EXPLANATION OF PROCEDURES

## INTRODUCTION

This is an outline of the plan for phase two of the New Zealand Very Low Birthweight Young Adult Follow Up Study. It gives a few more practical details than the approved Information Sheet you also have.

The purpose of this study is to gather information about health and developmental outcomes in young adulthood for New Zealand children born in 1986 who were born very early (premature) or of very low birthweight (<1500 grams) compared with children born close to their expected date ("controls"). There has been a little research done in this area in New Zealand. There is a great deal of interest around the world in these young adults as modern medicine means most very premature children now survive when that was not always so in the past. Our aim is to obtain information on the long term outcomes of prematurity or very low birthweight for New Zealand children.

Involvement in this study is completely voluntary and you are free to withdraw at any time.

## ANY QUESTIONS?

If you would like to talk to us about any parts of this study, please contact:

Julia Martin - Project Manager
03 378 6437
021 248 7999
julia.martin@otago.ac.nz

Professor Brian Darlow – Principal Investigator
Phone: (03) 3644699 Ask for pager: 5038
brian.darlow@otago.ac.nz

## OLD STUDIES AND NEW

You are one of just over 300 survivors from a total of 413 children who were born in New Zealand in 1986 weighing less than 1500 grams at birth – or have volunteered to be part of the study as a control. When you were aged 7-8 years, with your parent's permission you were involved in a study looking at developmental outcomes for very low birthweight infants. When you were 23-24 years, you were invited to undertake an interview with a research nurse on matters relating to your health and life experience. We are now inviting you to participate again and we are looking in more detail at health issues and will be undertaking a number of medical investigations and tests of ability and functioning that weren't undertaken last time.

## WHAT WILL BE INVOLVED?

The study will involve a trip to Christchurch for a two day series of investigations. We will pay for your accommodation, food and travel expenses. You will be reimbursed $200 to compensate you for any inconvenience or loss of earnings and in exchange for your participation. There are several parts to these two days of assessments – these are discussed further in this booklet.

## WHERE?

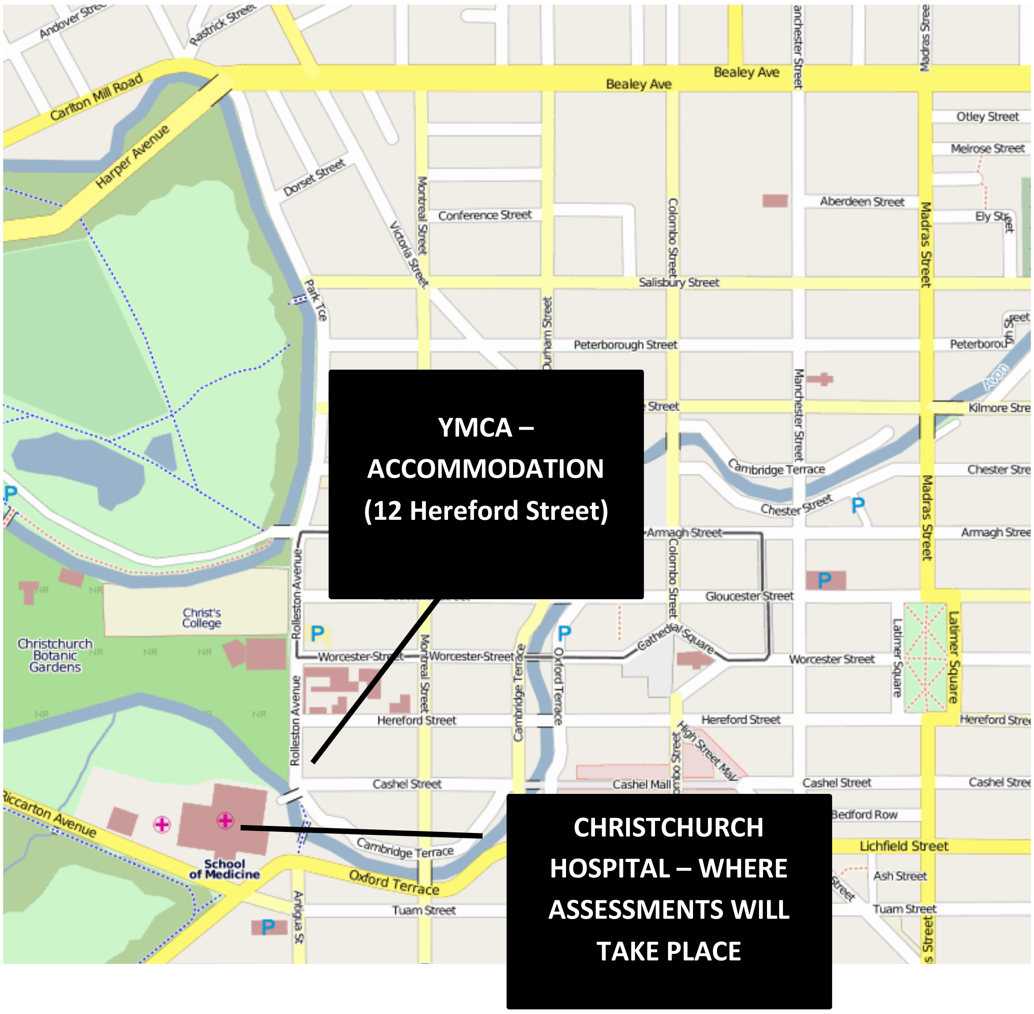

## WHAT KIND OF INTERVIEWS AND TESTS?

- **Medical investigations (1 – 1.5 hours)**

**Standard questionnaires:**

You will be asked a few standard questions about specific health issues, including respiratory (lung or breathing) and gastrointestinal (gut or bowels).

**Bloods Tests (70ml*):**

This is taken from a vein in your arm and can be a little uncomfortable for a short time. We can use a local anaesthetic spray if you prefer. There might be a small bruise afterwards. Some tests need to be "fasting" so the test will be taken before breakfast one day. (* If you agree to Genetic Tests, for which there is a separate information sheet and consent form, an extra 10mls of blood will be taken).

**Kidney function (including early morning urine):**

This is calculated from a blood test and from sampling the first urine specimen you pass in the morning.

**Growth (Height, Weight, Head Circumference, BMI, Waist and Hip measures):**

BMI is Body Mass Index and is calculated from height and weight measurements.

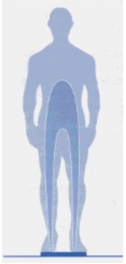

**Total body fat (bioelectrical impedance):**

Bioelectrical Impedance Analysis (BIA) is a method of screening body fat. Your height, age and gender are entered into the scales. You will stand on scales in bare feet with your hands at your sides. It sends a tiny, unnoticeable current up one leg and down the other. BIA is safe and it does not hurt. It takes less than a minute.

**Blood Pressure:**

This is taken with you sitting down and a cuff is inflated quite tightly around the arm. The average of three readings is taken.

**Endothelial dysfunction (EndoPAT):**

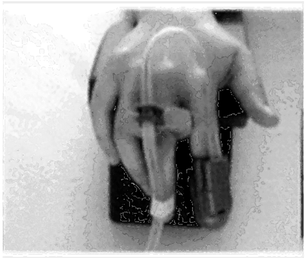

As with blood pressure, a cuff is inflated around the arm for several minutes so it might feel a bit uncomfortable. As the same time a thimble-like probe is worn on one finger of each hand (on the index finger in the picture). When the cuff is let down the probe measures the blood flow in the finger and how "flexible" the arteries are.

**ECG and Echocardiogram:**

The electrocardiograph is a device used to record on graph paper the electrical activity of the heart. The echocardiogram is an ultrasound examination of the heart.

These procedures are performed in a private part of the clinic by a senior cardiac sonographer and research nurse. You'll be asked to remove your clothing from the waist up. A drape/gown is provided for your privacy. The sonographer will place three electrodes (small, flat, sticky patches) on your chest. The electrodes are attached to an electrocardiograph (ECG) monitor that charts your heart's electrical activity during the test.

You will be asked to lie on your left side on an exam table. The sonographer will place a wand (called a sound-wave transducer) on several areas of your chest. The wand will have a small amount of gel on the end, which will not harm your skin. This gel helps produce clearer pictures. You may hear the sound of your heart beating during the test. You may be asked to change positions several times during the exam so the sonographer can take pictures of different areas of your heart. You may also be asked to hold your breath at times. After the test, more electrodes will be placed across your chest wall to record a full electrocardiograph (ECG) tracing. Both tests take about 30 minutes in total. You should feel no discomfort during the test. You may feel a coolness on your skin from the gel on the transducer, and a slight pressure of the transducer on your chest.

- **Visual assessments (0.5 hours)**

**Glasses prescription:**

Your glasses (if you wear them) are placed in a machine that can "read" the prescription.

**Distance visual acuity (ETDRS):**

You will be asked to read the letters on a standard chart (with glasses if you wear them).

**Contrast sensitivity (Pelli-Robson charts):**

Again reading letters on a chart but some are more sharp than others.

**Autorefraction:**

Drops are put into the eye to dilate the pupil for a short time. A camera-like device is then pointed at the eye to take measurements. It is quick, simple and painless.

**Retinal photographs:**

Very similar to autorefraction, except that a camera takes a photograph of the back of the eye.

- **Respiratory function tests (1 – 1.5 hours)**

Before this session you will be asked a few questions to make sure you can do all the tests. A respiratory scientist will then ask you to perform several breathing tests.

Please DO NOT USE ANY BREATHING INHALERS (relievers) on the day of your tests, but do bring them with you.

Please DO NOT SMOKE on the day of your tests.

**Spirometry:**

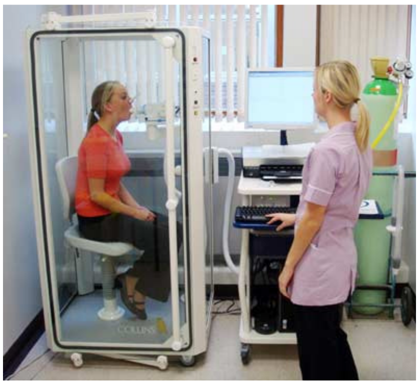

This involves taking a big breath in until you are full – then blowing all the air from your lungs as fast as possible until you are empty.

**Lung Volumes:**

For this test you will be seated in a clear cubicle with the door closed (see photo). You will be asked to breathe normally through the mouthpiece for a few breaths. We will then ask you to briefly pant against a blockage in the mouthpiece, followed by a breath in until full, followed by a breath out until you are empty.

**Diffusing Capacity:**

You will be instructed to blow out through the mouthpiece until empty, then take a large breath of test gas, hold your breath for 10 seconds, then breathe out.

**Single Breath Nitrogen Washout:**

We will ask you to breathe in a full breath of 100% oxygen and then breathe out until you are empty at a controlled rate.

**Exercise Test:**

This test involves exercising on a stationary cycle with an increasing workload for as long as you are able (approximately 8-12 minutes). During the test we will record your breathing and the amount of oxygen you use through a breathing mask. We will place some ECG patches on your chest to monitor your heart (see photo). A doctor will be present during the exercise test.

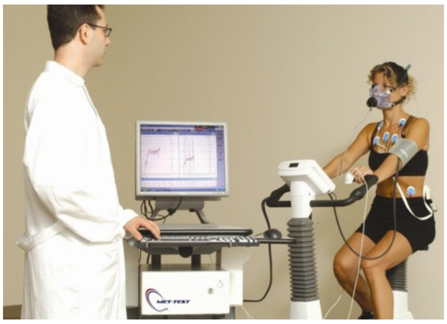

- **Cognitive and Neuropsychological functioning (2-3 hours)**

These tests will all be done in a quiet room, sitting at either a desk or a computer screen. The test supervisor will be an experienced psychologist. Some tests involve answering questions and others are a bit more like games or puzzles. Some look at the sort of tasks we undertake everyday. Most people find the tasks interesting and even fun. There will be time for a drink break during the tests.

- **Health and Social functioning questionnaire (1-1.5 hours)**

This will be a face-to-face interview. As at 23-24 years, questions will cover a range of topics including your education, employment, general health, social relationships and related matters.

- **Magnetic Resonance Imaging - MRI (1 hour)**

**Note:** Due to MRI testing being very expensive only 150 participants and 50 controls will participate in this. You will be randomly selected.

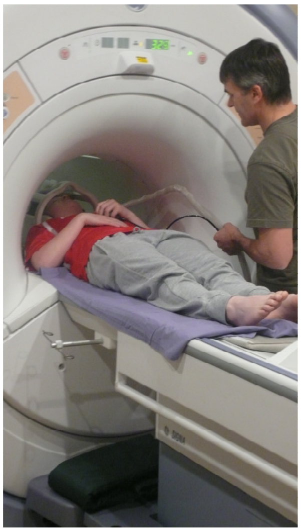

Magnetic Resonance Imaging or MRI is a powerful, non-invasive imaging technique used in medicine to investigate the interior of the human body and brain. MRI has revolutionized medical imaging by providing unique contrast between soft tissues of different types. MRI uses no damaging (ionizing) radiation, unlike other useful imaging techniques like x-ray and Computed Tomography (CT), but instead exploits the magnetic properties of tissue which gives better quality information.

All imaging will occur at the **Hagley Radiology, 16 St Asaph Street, Christchurch**. The MRI scanning session will last approximately 45 minutes. During this time, we will acquire a number of different MR images, using a variety of clinical techniques. Imaging is completely non-invasive, safe, and painless. Upon arrival participants will go through a rigorous safety check to ensure that no metal enters the scanning room (the MRI scanner is a large magnet and therefore attracts coins and other metal and can interfere with pacemakers or neurostimulators). Participants will lie down inside the scanner and will be asked to keep as still as possible. When acquiring images, the MRI scanner is moderately noisy. Participants will therefore receive ear protection (earplugs and/or earphones). It is even possible to listen to music during approximately half the scanning session.

We will assess the overall appearance of the brain and how different areas work together.

**Benefits and Risks:** The MRI scans are totally non-invasive and painless. It is possible that some participants may feel uncomfortable being in the confined space of the MRI unit (but no more than 5% will experience these feelings). If there is any case where a participant feels distressed, fatigued, or claustrophobic during the scanning session, it will be terminated immediately.

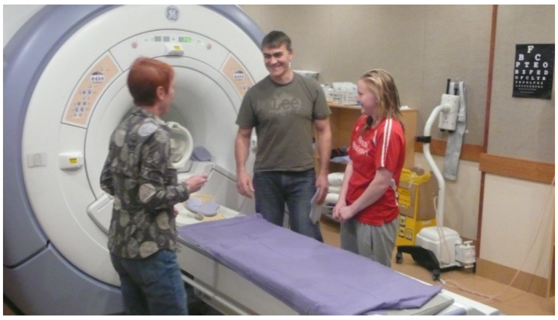

At the end of the session we will give you a DVD with some of the brain images on it.

- **Dental Examination (45 minutes)**

Dental examinations will take place at the Oral Health Centre, 2 Riccarton Ave.

During this time, the dentist will ask some questions about any medical conditions that may affect your dental check-up, and about previous orthodontic (tooth straightening) treatment. They will check the soft tissues in the mouth (lips, gums, tongue, palate, floor of mouth), and record how many teeth you have and whether there are any missing teeth. If you wear false teeth (denture or partial plate) the dentist will look at them as well.

A dental probe will be used to gently check the health of your gums, and can sometimes feel a little uncomfortable for a short time. The dentist will then check your teeth to record any fillings that you have had done, whether there are any cavities (tooth decay), and whether there are any defects in the structure of the tooth enamel.

## THE INFORMATION SHEETS and CONSENT FORM

The approved Information Sheet gives full details of the study and the Consent Form contains a list of all the major sections planned for phase two of the NZ VLBW follow up study. This will be discussed and signed when the participant meets with the Project Manager.

## CONFIDENTIALITY

We fully appreciate that the information you provide is personal and we would advise you that all material that you provide us will be treated in the utmost confidence. Your identity will not be revealed in any reports based on this study. Other research groups may be supplied with data from the NZ very low birthweight study but in all cases this information will be anonymous.

## SECURITY OF INFORMATION

All information collected by the NZ VLBW study is kept secure. Information is linked to anonymous numbers and special security procedures are in place to prevent the information being linked to the names of study members. No material which could identify any individual is ever used in any reports of the study.

## PARTICIPANT FEEDBACK

We will provide you with a summary of your findings. Should we identify any clinically significant abnormal results, we undertake, with your permission, to make these known to your GP, who can then explain them to you.

**PLEASE REMEMBER THE FOLLOWING**

- Bring along any medication you are currently taking
- Bring along your current GP and dentist name/contact details
- Bring along contact details of your family/people we can contact if you move
- Wear comfortable/loose clothing
- If you wear glasses please bring them with you and if you wear contact lenses please bring your prescription
- Please also tell us – in confidence – if you are pregnant because this may mean you should not participate in some of the tests.
- Bring your bank account details – so we can transfer the $200 into your account

**THANKS FOR PARTICIPATING IN THIS STUDY - WE REALLY APPRECIATE IT**
